# Sediment Properties as Important Predictors of Carbon Storage in *Zostera marina* Meadows: A Comparison of Four European Areas

**DOI:** 10.1371/journal.pone.0167493

**Published:** 2016-12-09

**Authors:** Martin Dahl, Diana Deyanova, Silvia Gütschow, Maria E. Asplund, Liberatus D. Lyimo, Ventzislav Karamfilov, Rui Santos, Mats Björk, Martin Gullström

**Affiliations:** 1 Seagrass Ecology & Physiology Research Group, Department of Ecology, Environment and Plant Sciences, Stockholm University, Stockholm, Sweden; 2 The Sven Lovén Center for Marine Sciences, University of Gothenburg, Fiskebäckskil, Sweden; 3 School of Biological Science, University of Dodoma, Dodoma, Tanzania; 4 Institute for Biodiversity and Ecosystem Research at the Bulgarian Academy of Sciences, Sofia, Bulgaria; 5 ALGAE -Marine Ecology Research Group, CCMar - Center of Marine Sciences, University of Algarve, Faro, Portugal; Beijing Normal University, CHINA

## Abstract

Seagrass ecosystems are important natural carbon sinks but their efficiency varies greatly depending on species composition and environmental conditions. What causes this variation is not fully known and could have important implications for management and protection of the seagrass habitat to continue to act as a natural carbon sink. Here, we assessed sedimentary organic carbon in *Zostera marina* meadows (and adjacent unvegetated sediment) in four distinct areas of Europe (Gullmar Fjord on the Swedish Skagerrak coast, Askö in the Baltic Sea, Sozopol in the Black Sea and Ria Formosa in southern Portugal) down to ~35 cm depth. We also tested how sedimentary organic carbon in *Z*. *marina* meadows relates to different sediment characteristics, a range of seagrass-associated variables and water depth. The seagrass carbon storage varied greatly among areas, with an average organic carbon content ranging from 2.79 ± 0.50% in the Gullmar Fjord to 0.17 ± 0.02% in the area of Sozopol. We found that a high proportion of fine grain size, high porosity and low density of the sediment is strongly related to high carbon content in *Z*. *marina* sediment. We suggest that sediment properties should be included as an important factor when evaluating high priority areas in management of *Z*. *marina* generated carbon sinks.

## Introduction

Seagrass ecosystems are considered highly efficient natural carbon sinks [1] but there is a large variation in their capacity to store carbon, depending on species composition and habitat characteristics [2,3]. While the carbon sequestration efficiency is quite well documented for many seagrass species (e.g. [4,5]) the effects of different factors influencing intraspecific variation has only recently been investigated. To get a more accurate estimate of the global seagrass carbon sink capacity cause-effect relationships need to be better understood, and as seagrass loss is accelerating [6] information on habitat characteristics affecting carbon storage are of importance for an efficient protection and management strategy to increase carbon storage capacity [7].

There are several environmental factors (e.g. water depth and hydrodynamic processes) and seagrass habitat variables (e.g. canopy height and shoot density) that influence the carbon storage in seagrass sediments [8]. For example, seagrass meadows at shallower depths are known to have a high accumulation of sedimentary carbon [9], which could be associated with higher primary production and larger standing biomass stock [10]. Dense meadows have the ability to stabilize the sediment (and thereby preventing it from eroding) [11] and seagrass habitats with a high canopy can trap a high amount of suspended particles and thus potentially increase the sedimentation of organic matter [12,13]. Further, as the belowground biomass largely contributes to the carbon storage due to its high production, fast turnover and higher decay-resistant lignin content compared to the leaves [14,15] a large root-rhizome system could render a higher carbon storage [16]. In the coastal environment, sediment grain size is known to influence the aggregation of organic particles with finer grain sizes increasing the organic matter content of the sediment [17]. By reducing water velocity and facilitating sedimentation processes a seagrass meadow could increase the amount of fine particles, which thus promote high carbon storage. Grain size has recently been shown to correlate with sedimentary carbon content in some seagrass areas [18,19], especially in meadows with a low contribution of autochthonous derived carbon, although the influence of grain size on carbon storage is not universal for all seagrass species and habitats [19]. Grain size is also strongly related to sediment porosity and density, which are important factors influencing the oxygen conditions in the sediment. Oxygen levels together with the microbial community composition, water temperature, biomass carbon and nutrient content are important factors for the degradation rate of organic matter in the sediment [20–23] and therefore influencing the carbon sequestration process.

*Zostera marina* L. is the most widely spread seagrass species in the northern hemisphere, with a distribution in Europe stretching from the southern Black Sea and the Gulf of Cádiz (southern Portugal) up to Iceland and the northern parts of Norway [24]. The plant biomass is generally larger at higher latitudes [25] because of more optimal growth temperatures [26]. Large seagrass populations can be found along the Swedish west coast and at the east coast of Denmark [27,28], where they form extensive meadows with shoots over 1 m in length. The species can tolerate salinity ranging from 5 to 35 [29] and a depth distribution from the intertidal down to 30 m depending on water clarity [30]. *Zostera marina* also grows in various substrates, from coarser stone-sand bottoms to finer silt and clay sediment. In this study, we aim to assess and compare carbon storage in *Z*. *marina* meadows at four different areas in Europe as well as to examine relationships between sediment organic carbon content and several explanatory variables including water depth, seagrass structural complexity, carbon and nitrogen content of the seagrass biomass and sediment characteristics (i.e. sediment porosity, density and grain size) in order to determine factors influencing the storage capacity of *Z*. *marina* meadows in these areas.

## Methods

### Study sites

**Fig 1:**
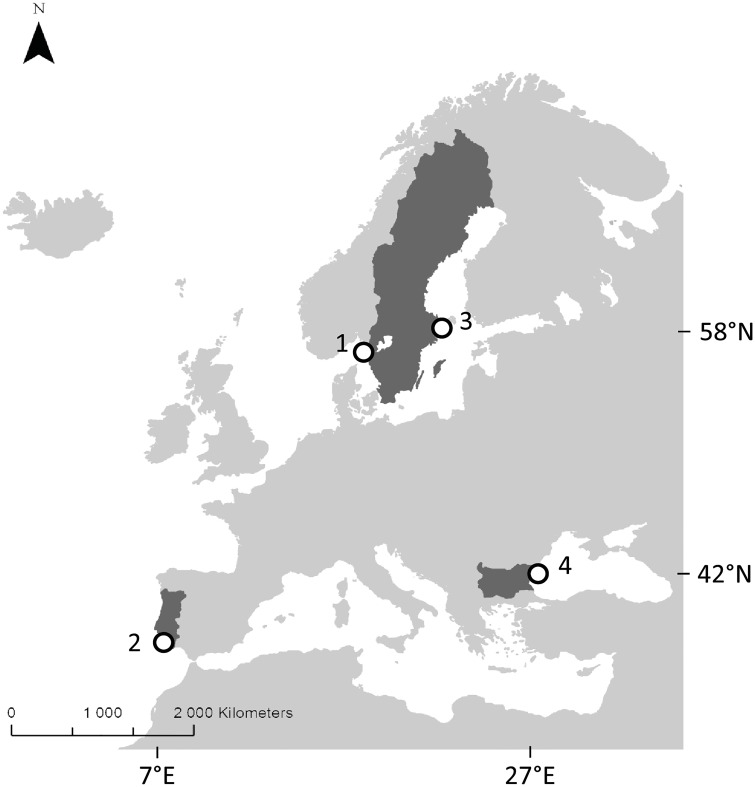
Map showing the study areas. The four study areas include the Gullmar Fjord (Skagerrak, Sweden) (1), Ria Formosa (Gulf of Cádiz, Portugal) (2), Askö (Baltic Sea, Sweden) (3) and Sozopol (Black Sea, Bulgaria) (4). The map is adapted from Esri ArcGIS online basemaps.

For sampling seagrass and sediment on the study sites no permission was required according to the countries’ national legislations and no protected species was part of this study as *Z*. *marina* is not on the IUCN list of endangered species. This study was conducted in four different areas in Europe (the Swedish Skagerrak and Baltic coasts, Black Sea in Bulgaria and the southern coast of Portugal; Fig 1, Table 1) from June to October 2013 with one complimentary field sampling performed in October 2014. The different study areas cover a range of environmental and physical conditions (e.g. salinity and water temperature) for *Z*. *marina* in Europe. In each area, sampling was conducted in two meadows and one unvegetated area (reference site). Additionally, in Portugal one unvegetated area was added and in the Baltic Sea one meadow and one unvegetated area were added (Table 1). The growth season for *Z*. *marina* and the annual water temperature in the different areas vary due to latitude. On the Swedish Skagerrak coast, the growth season stretches from May to November with a peak in August [31], and with an annual water temperature ranging from 0 to 25°C [27]. In the Baltic Sea, the growth season stretches from May to October [32], and with an annual water temperature ranging from 0 to 22°C [33]. The peak of the growth season in the Black Sea is between May and July depending on previous winter conditions (Karamfilov pers. com), and in Ria Formosa the growth season peak is in June-July [34]. The water temperature in the Sozopol area is between 5 and 29°C [35], while in Ria Formosa the temperature ranges from 12 to 27°C [36]. The sampling on the Swedish west coast was carried out in June 2013 off The Sven Lovén Centre for Marine Sciences—Kristineberg in the Gullmar Fjord (58°20’N, 11°33’E; Table 1). The area is comprised of small islands and shallow bays making it highly productive and a suitable environment for seagrass growth with many sheltered soft bottoms covered by extended *Z*. *marina* beds. Seagrass meadows at the Swedish west coast are known to have existed for a long time with reports dating back to the 1880s [37] and detailed distribution data for the specific study sites reported from the 1980s [38,39]. In the Baltic Sea, samples were collected in the area around the Askö Laboratory in the Stockholm Archipelago (58°49’N, 17°39’E) in October 2013 and 2014. The Baltic Sea is a brackish water system and the salinity is about 5–6 outside Askö, which is on the distribution limit for *Z*. *marina* [29]. Low salinity is known to negatively affect production and growth of the plant [40]. The growth rate of *Z*. *marina* is 1.5 g dw m^-2^ d^-1^ in the Baltic Sea compared the higher growth rate of 3.5 g dw m^-2^ d^-1^ on the Swedish Skagerrak coast [41]). In the Baltic Sea, *Z*. *marina* grows at approximately 2–5 m depths (sometimes together with *Ruppia maritima*) and on more coarse sediment compared to the Skagerrak area [27]. *Zostera marina* in the Baltic Sea has been shown to be very old (potentially >1000 years) due to clonal horizontal rhizome growth [42], while in the study area colonization data is lacking but existence of seagrass meadows was reported in the 1970s [43]. In the Black Sea, sampling was carried out in June 2013 at two sites around the Laboratory of Marine Ecology in Sozopol, Bulgaria (42°25’N, 27°41E). The salinity in the area is around 17 and commonly *Z*. *marina* grows in mixed stands with *Z*. *noltii*. The first survey on *Z*. *marina* along the Bulgarian coast was carried out in 1977–78, in which our study sites were reported, and the estimates of biomass were similar to those found in recent years (2010–11) indicating that the seagrass meadows have been stable for many years [35,44]. The sampling in Rio Formosa (Algarve Marine Sciences Centre—Faro) took place in August 2013. Ria Formosa is located in southern Portugal (36°59’N, 7°52’W) and is a coastal lagoon with large intertidal areas and a tidal fluctuation of 2–3 m. This is the only area in the present study with pronounced tidal variation, and the water depth for the Portugal sites was standardized to mean low water (MLW) by calculating the difference between the measured water depth and the tide at the time of measurement. The tide values were obtained from the Ria Formosa tidal station (Faro-Olhão) with the mean water level as reference depth. The first observation of *Z*. *marina* in our study sites (around Culatra Island) was done in 1991 [45,46]; however, the presence of *Z*. *marina* in Ria Formosa was already reported by den Hartog [47] and is most likely not a recently introduced species to the area. Today, the distribution of *Z*. *marina* (which at times grows together with *Cymodocea nodosa*) is scarce and apart from one other area in Portugal (Óbidos Lagoon) the only one that still harbor *Z*. *marina*, which has decreased drastically during the past 20 years [46].

**Table 1:** Description of study sites in the four areas of Europe.

Area	Site	Vegetation	Coordinates	Mean depth (m)
Gullmar Fjord (Skagerrak, Sweden)				
	Finnsbo (F)	*Z marina*	58°17'55N, 11°29'34E	2.8
	Kristineberg (K)	*Z*. *marina*	58°14'53N, 11°26'51E	3.0
	Rödberget (Rö) (r)	Unvegetated	58°15'06N, 11°27'54E	2.5
Ria Formosa (Gulf of Cádiz, Portugal)^1^				
	Culatra channel (C)	*Z*. *marina/ C*. *nodosa*	37°00'14N 7°49'36W	1.9
	Ilha da Culatra (I)	*Z*. *marina*	36°59'50N, 7°49'41W	1.0
	Culatra channel (Cr) (r)	Unvegetated	37°00'15N, 7°49'33W	2.6
	Ilha da Culatra (Ir) (r)	Unvegetated	36°59'51N, 7°49'40W	1.8
Askö (Baltic Sea, Sweden)				
	Torö (T)	*Z*. *marina/R*. *maitima*	58°48'14N, 17°47'32E	3.2
	Långskär (L)	*Z*. *marina/R*. *maitima*	58°48'00N, 17°40'48E	2.2
	Storsand (S)	*Z*. *marina*	58°48'26N, 17°41'40E	3.8
	Torö (Tr) (r)	Unvegetated	58°48'21N, 17°47'31E	6
	Godahoppsudden (Gh) (r)	Unvegetated	58°48'09N, 17°42'24E	2.9
Sozopol (Black Sea, Bulgaria)				
	Ropotamo (Rt)	*Z*. *marina*/ *Z*. *noltii*	42°19'49N, 27°45'20E	2.7
	Gradina (G)	*Z*. *marina*/ *Z*. *noltii*	42°25'39N, 27°39'05E	4.2
	Bay of Sozopol (r)	Unvegetated	42°24'42N, 27°39'48E	5.7

r = reference site (unvegetated area)

^1^Depth values standardized to mean low water (MLW).

### Sediment sampling and biometrical measurements

At each site, six sediment cores were taken with a push corer (h = 50 cm, ø = 8 cm) at a distance of 10–30 m apart from each other. The edge of the corer was sharpened to easier press down the core into the sediment and to reduce the shortening (compression) of the sediment collected [48]. However, due to the difference in sediment compactness among sites the length of the sediment core varied (because of difficulties in pressing down the core in coarser sediment). Each core was sliced into a maximum of six depth segments (0–2.5 cm, 2.5–5 cm, 5–12.5 cm, 12.5–25 cm, 25–37.5 cm, 37.5–45 cm) with the majority of samples lacking the deepest segment. The corers were stored vertical prior to slicing the sediment into the different segments. We examined the influence of core shortening in the Skagerrak area, where the compression is expected to be the highest in our study due to the soft sediment and high porosity [49], by measuring outer and inner length of the corer to the sediment surface (n = 6). The effect of core shortening was derived from the difference between the inner and outer length of the corer and compression was calculated to be 8%. This has not been corrected for in the data and is further addressed in the discussion as a source of error. Within a few meters from each core at the seagrass sites, shoot height (cm, n = 20) was measured, percentage seagrass coverage (n = 10) was estimated (in 0.5 x 0.5 m squares) and biomass samples (n = 3) were collected (0.25 x 0.25 m). The biomass samples were used for estimating above- and belowground seagrass biomass (as dry weight) and for counting number of shoots. Before weighing the seagrass was cleaned and epiphytes removed, and the dry weight was measured after 24–48 h in 60°C until constant weight. One out of the three biomass samples collected around each core was analyzed for carbon and nitrogen content (n = 6 for each meadow). The sediment samples were cleaned from roots and rhizomes, larger shells and benthic organisms, and homogenized prior of drying. The sediment was dried in 60°C for approximately 48 h until the weight was constant. Before drying a sediment sample it was divided into two subsamples, one for analysis of carbon and nitrogen content, and the other for grain size analysis. A mixing mill (Retch 400 mm) was used to grind the sediment into a fine powder to further homogenize the subsample used for analysis of carbon and nitrogen content. The carbon and nitrogen contents in biomass and sediment were analyzed using an organic elemental analyzer (Flash 2000, Thermo Fischer scientific). Prior to analysis for organic carbon content the sediment samples were pre-treated with 1 M HCl (direct addition until the reaction of carbonate was complete) to remove inorganic carbon and dried at 60°C for 24 h. Total nitrogen was derived from untreated sediment samples due to possible alteration of the nitrogen values when treated with HCl [50]. Sediment porosity was given as percentage (%) by calculating sediment wet weight minus dry weight divided by the sample volume, whereas sediment density (g DW mL^-1^) was derived from dividing the dry weight of the sediment by the volume of the sample. A literature survey for measurements of sediment carbon content in *Z*. *marina* meadows in Europe and other temperate regions was conducted using Web of Science and Google Scholar with the search words “*Zostera marina*, sediment, organic”. Additionally, grey literature including thesis work was also used as well as unpublished data from colleagues.

### Grain size analysis

Three sediment cores in each habitat were used for grain size analysis and each depth section was separately analyzed. Prior to analysis the total dry weight of sediment for each section was determined (the average weight of the samples was 97 g) and 100 ml of 0.05 M Na_4_P_2_O_7_ was added to break down aggregates of clay particles. All of the sediment samples were dry-sieved for 10 min using a sieving tower (CISA electromagnetic sieve shaker, Spain) (including sieves of 0.074 mm, 0.125 mm, 0.25 mm, 0.5 mm, 1 mm and 2 mm) and the sediment of each sieve was weighed to determine the weight of the separate fractions. In depth sections with high organic carbon content (> 0.5%), the organic matter was removed prior to dry sieving, through oxidation with 35% H_2_O_2_, as the organic matter content leads to aggregation of particles [51]. When the reaction with H_2_O_2_ had ceased the samples were centrifuged for a minimum of 20 min at 4500 RPM, in which the supernatant was carefully removed using a pipette, and subsequently the samples were washed in distilled water and centrifuged again to remove H_2_O_2_ residues. After dry seiving, some of the samples from the Skagerrak and Ria Formosa areas had to be analysed with hydrometer for an accurate estimate of total grain size due to a high proportion of finer fractions (> 15% was assessed as < 0.074 mm) in those sediments. The samples were once more treated with 0.05 M Na_4_P_2_O_7_ and placed in a 1L cylinder containing distilled water and kept in suspension. At fixed time intervals (1, 2, 4, 10, 20, 50, 100, 200, 400 and 1000 min) the hydrometer was inserted and the concentration of sediment (g L^-1^) was noted. The mean grain size was presented in phi (ɸ) units.

### Statistical analysis

To test for differences in sedimentary carbon storage (% C_org_ and g C_org_ cm^-2^) and grain size particles < 0.074 mm (%) among areas, between *Z*. *marina* and unvegetated areas (habitat) and among sediment depths, nested general linear mixed model ANOVAs were performed using site as random factor and with habitat nested in area and sediment depth nested in core. In those cases where the ANOVA models were significant, Tukey’s HSD post hoc test was used to determine significant differences between specific areas and between habitats (*Z*. *marina* meadows vs. unvegetated areas). Prior to analysis all data were checked for normal distribution using the Shapiro-Wilk normality test and homogeneity of variances using Levene’s test. When assumptions were not met the data was log_10_(x+1) transformed. Partial Least Square (PLS) regression technique (by modeling of projections of latent structures [52] and Principal Component Analysis (PCA) were conducted in SIMCA 13.0.3 (UMETRICS) to test the influence of sediment characteristics, water depth and seagrass-related variables on sediment carbon content (mean % C for the top 25 cm of sediment). The advantage of using PLS modeling is that it can handle collinear explanatory data as well as a large number of predictors. All cores were standardized to a depth of 25 cm for the sediment characteristics (porosity, density, grain size and organic carbon content) prior to the PLS- and PCA analyses. Some of the cores at Askö (both seagrass- and unvegetated sites) lacked the 12.5–25 cm depth segment and in these cases logarithmic regressions were used (Eqs 1–4) to extrapolate the data down to 25 cm depth.

Torö (T) % C_org_

y=−0.87ln(x)+0.3845 (1)

Torö (T) g C_org_ cm^-2^

y=−0.001ln(x)+0.0052 (2)

Torö (Tr) (r) % C_org_

y=0.032ln(x)+0.2225 (3)

Torö (Tr) (r) g C_org_ cm^-2^

y=−0.0002ln(x)+0.0053 (4)

The carbon content in seagrass meadows decreases logarithmically with sediment depth in general [5] due to degradation and remineralization of organic material with time [53,54].

## Results

### Variation in sedimentary carbon storage

**Fig 2:**
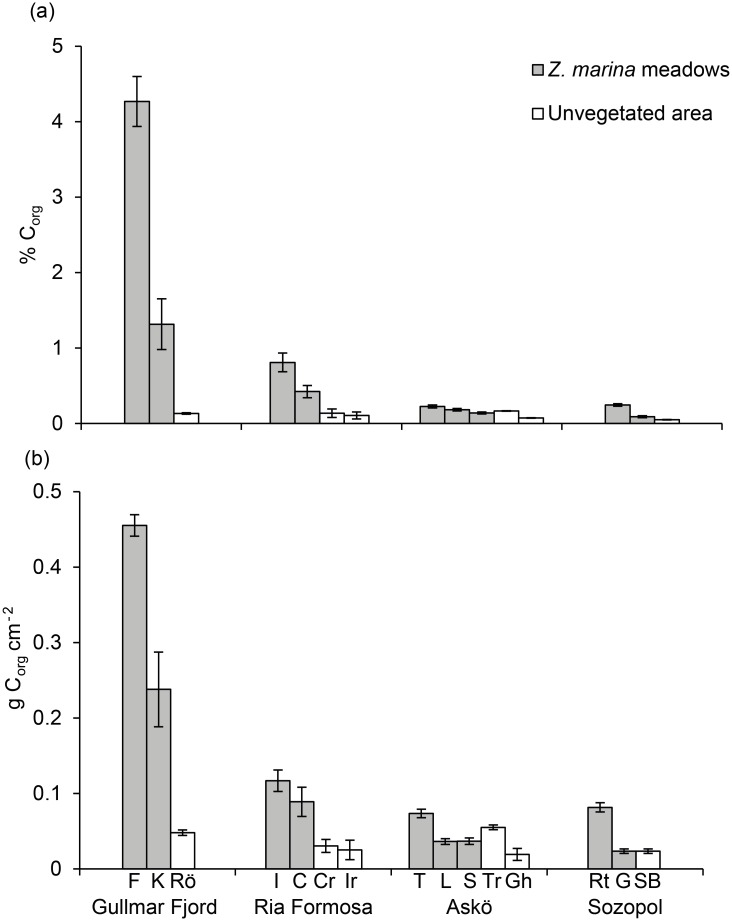
Sedimentary organic carbon content in *Z*. *marina* meadows and unvegetated areas. Mean (±SE) percent organic carbon (% C_org_) (a) and g C_org_ cm^-2^ (b) in sediment (for 0–25 cm sediment depth). The % C_org_ is presented as a mean of the content for the top 25 cm sediment, while carbon per unit area (g C_org_ cm^-2^) is the total (accumulated) amount of carbon in the top 25 cm of sediment. For full names of the sites see Table 1.

**Fig 3:**
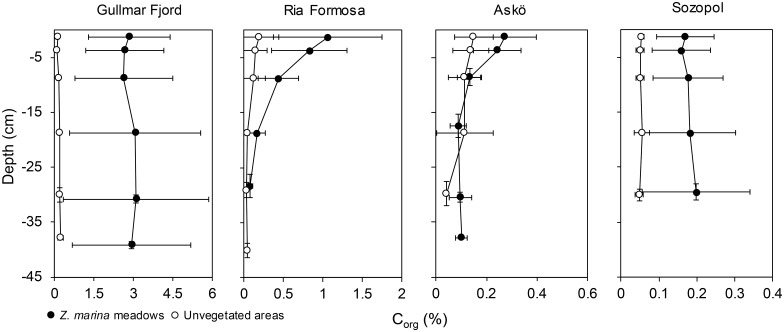
Organic carbon depth profiles. Mean sedimentary carbon (% C_org_ ± SD) depth profiles grouped for the different areas showed as mean slice depth. Note that the scale on the x-axes vary among the different depth profiles due to large variation in carbon content among areas.

The *Z*. *marina* meadows had significantly higher sedimentary carbon content compared to the unvegetated areas (*P* < 0.001, Table 2). Only the Gullmar Fjord and Ria Formosa, however, showed significantly different values in *Z*. *marina* compared to their respective unvegetated areas (*P* < 0.001), while Askö and Sozopol did not show any between-habitat differences (Fig 2). The Gullmar Fjord was significantly different from all other areas (*P* < 0.05), whereas Ria Formosa was significantly different to Sozopol (*P* < 0.05) but not to Askö, and no difference was seen between Sozopol and Askö (Fig 2). The highest amount of sedimentary carbon was seen in the Gullmar Fjord, followed by Ria Formosa, Askö and Sozopol (Fig 2, Table 3). There were no significant differences in either % C_org_ or g C_org_ cm^-2^ among the different sediment depths (Fig 3, Table 2).

**Table 2:** Summary of nested general linear mixed model ANOVAs for sediment carbon content and sediment grain size. The factor habitat is comparing *Z*. *marina* meadows and unvegetated areas. Bold values indicates significant values (*P* < 0.05).

Source of variation		% C_org_			g C_org_ cm^-2^			Grain size (< 0.074 mm, %)		
	df	MS	*F*	p	MS	*F*	p	MS	*F*	P
Area	3	1.0734	81.00	**< 0.001**	0.000	98.36	**< 0.001**	4.6387	78.32	**< 0.001**
Habitat (Area)	4	1.1603	87.55	**< 0.001**	0.000	112.23	**< 0.001**	4.7868	80.82	**< 0.001**
Core	5	0.0188	1.42	0.218	0.000	2.30	**0.045**	0.0415	0.70	0.498
Sediment depth (Core)	24	0.0107	0.81	0.724	0.000	1.51	0.059	0.0677	1.14	0.328
Residual	378	0.0133			0.000			0.0592		

**Table 3:** Seagrass sediment data. Values are presented as mean (± SD for all variables except carbon content, which is presented with ± SE) for the depth profiles (0–25 cm) in the different areas. Mean grain size is presented with phi (ɸ) units.

Areas	% C_org_	g C_org_ cm^-2^	Sediment porosity (%)	Sediment density (g DW mL^-1^)	% N	C:N	Mean grain size (ɸ)	Sediment particles < 0.074 mm (%)
Gullmar Fjord	2.79 ± 0.50	0.35 ± 0.041	67.0 ± 14.1	0.71 ± 0.33	0.28 ± 0.16	9.39 ± 1.26	4.89 ± 0.93	62.8 ± 25.6
Ria Formosa	0.61 ± 0.09	0.10 ± 0.012	43.0 ± 5.4	1.13 ± 0.14	0.08 ± 0.01	7.15 ± 0.83	2.34 ± 0.56	17.9 ± 5.8
Askö	0.18 ± 0.01	0.05 ± 0.005	31.9 ± 2.4	1.4 ± 0.15	0.03 ± 0.01	5.60 ± 1.27	1.19 ± 0.79	3.7 ± 0.6
Sozopol	0.17 ± 0.02	0.05 ± 0.009	41.8 ± 5.2	1.25 ± 0.04	0.05 ± 0.04	3.22 ± 1.25	2.08 ± 0.27	2.6 ± 1.9

### Influence of sediment characteristics and seagrass-associated variables on carbon storage

**Fig 4:**
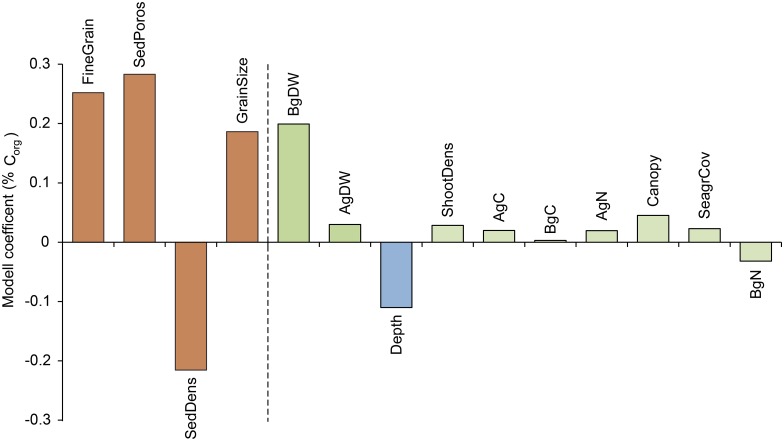
Partial least square (PLS) regression model coefficient plot. The model assesses the relative influence of different predictors on % C_org_ in sediment (using a mean for the top 25 cm sediment). The predictor variables are ranked in level of importance (left to right) where the four variables left of the striped bar having a VIP-value > 1 (i.e. FineGrain, SedPoros, SedDens and GrainSize) and hence significantly influencing % C_org_. Brown bars = sediment characteristics, green bars = seagrass-associated variables and blue bars = water depth. Variables included in the model were FineGrain (sediment particles < 0.074 mm, %), SedPoros (sediment porosity, %), SedDens (sediment density, g DW mL^-1^), GrainSize (mean grain size, ɸ), Bg and Ag DW (belowground [roots and rhizomes] and aboveground [shoots] biomass dry weight, g m^-2^), Depth (water depth, m), ShootDens (shoot density, shoots m^-2^), Ag and Bg biomass C and N (biomass carbon and nitrogen content, %), Canopy (shoot height, cm) and SeagrCov (seagrass cover, %).

When the relationship between % C_org_ and explanatory variables (Tables 1, 3 and 4) was examined in a PLS (Partial least square) regression model the sediment characteristics explained most of the model (with a variance of importance value > 1) where the proportion of sediment particles < 0.074 mm (%) was the most important variable, followed by sediment porosity (%), sediment density (g DW mL^-1^) and mean grain size (ɸ) (Fig 4, S1 Fig). These variables—characterizing the sediment—were all positively correlated to % C_org_ except sediment density that showed a negative relationship with % C_org_. The cumulative fraction explaining the % C_org_ variation (R_y_^2^ cum) of the predictor variables combined was 0.81 and the models cross-validated variance (Q^2^ statistics) showed high predictability with Q^2^-value of 0.79, thus larger than the significant level of 0.05. The results of the model with g C_org_ cm^-2^ is not shown here as the results (Q^2^ = 0.77, R_y_^2^ cum = 0.78) were highly similar to the results of % C_org_, with the same predictor variables (i.e. sediment characteristics) explaining most of the variation and being correlated in the same way. All seagrass-associated variables showed a positive relationship with % C_org_ except for nitrogen content in the belowground biomass (BgN, %), which was the least influential variable in the model. In general, the seagrass-associated variables showed a lower contribution to the overall model compared to the variables characterizing the sediment. Water depth (m) was negatively correlated to % C_org_ but was of minor importance for the overall model (Fig 4).

**Table 4:** Seagrass meadow variables (mean ± SD) for the different areas.

	Shoot density (m^-2^)	Shoot height (cm)	Seagrass cover (%)	Aboveground biomass				Belowground biomass			
Areas				% C	% N	C:N	g DW m^-2^	% C	% N	C:N	g DW m^-2^
Gullmar Fjord	157.9 ± 43.8	81.4 ± 18.2	36.9 ± 14.0	38.8 ± 0.7	2.1 ± 0.3	18.4 ± 2.0	39.4 ± 31.1	34.2 ± 1.4	1.1 ± 0.1	30.2 ± 2.8	253.0 ± 86.0
Ria Formosa	264.9 ± 107.8	32.5 ± 4.4	79.1 ± 10.6	34.4 ± 1.2	1.4 ± 0.1	25.2 ± 1.2	108.3 ± 58.5	30.8 ± 3.2	1.1 ± 0.1	37.8 ± 4.5	494.2 ± 230.2
Askö	338.1 ± 160.3	51.7 ± 12.4	47.6 ± 18.1	37.2 ± 2.0	1.8 ± 0.3	20.9 ± 2.9	255.7 ± 193.4	32.8 ± 2.9	1.1 ± 0.1	31.5 ± 3.2	205.9 ± 88.7
Sozopol	419.6 ± 315.3	63.5 ± 11.2	63.5 ± 11.1	36.0 ± 1.3	1.9 ± 0.3	19.6 ± 3.2	122.0 ± 110.8	30.3 ± 3.0	0.8 ± 0.2	28.6 ± 5.7	86.4 ± 80.6

**Fig 5:**
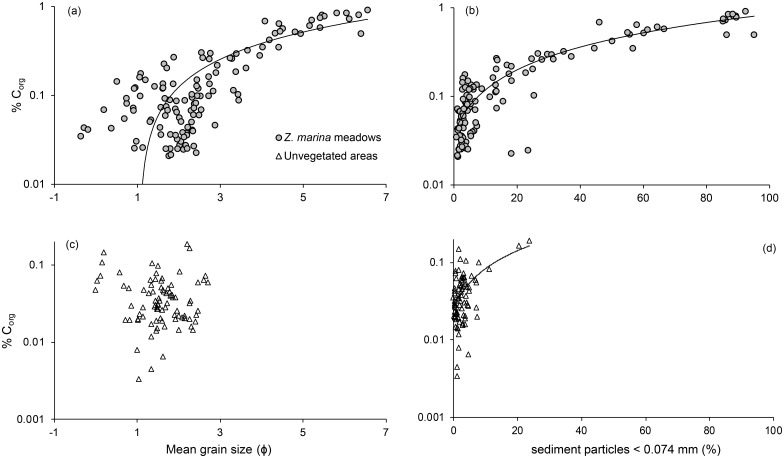
Semi-log plots (log_10_[x+1]) showing the relationship between sedimentary % C_org_ and grain size. The % C_org_ is presented with a log scale as it gave the best fit of the models. Grain size is shown as mean grain size (ɸ) and sediment particles < 0.074 mm (%) for *Z*. *marina* meadows (a and b) and unvegetated areas (c and d). The % C_org_ was positively linked to both sediment particles < 0.074 mm (%) (R^2^ = 0.91, *P* < 0.001) and mean grain size (ɸ) (R^2^ = 0.74, *P* < 0.001) for *Z*. *marina* meadows but for unvegetated areas only sediment particles < 0.074 mm (%) showed such relationship with % C_org_ (R^2^ = 0.42, *P* < 0.001).

**Fig 6:**
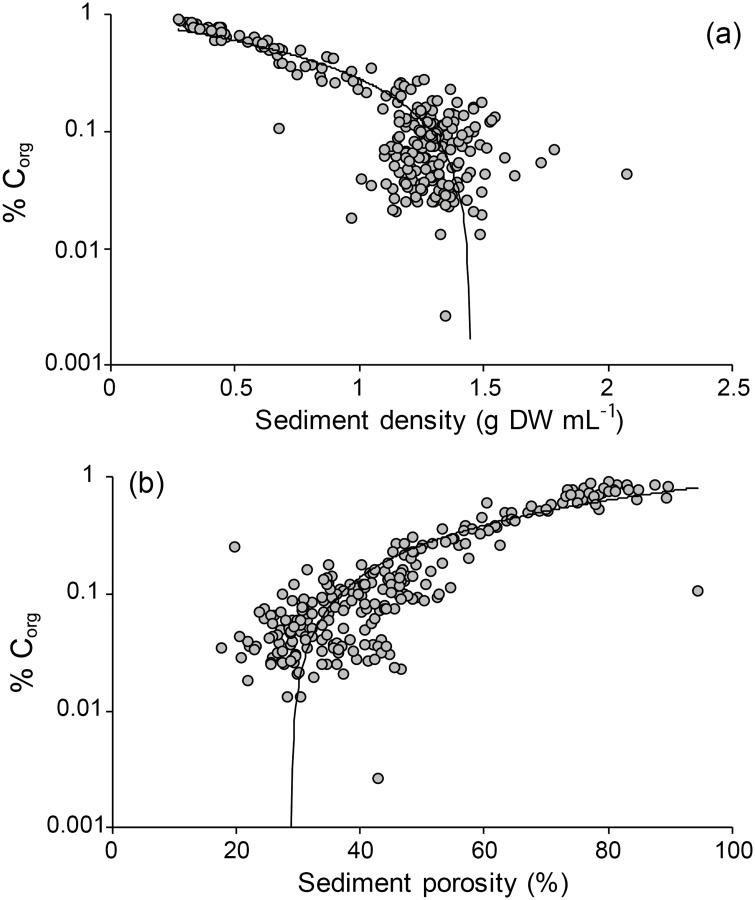
Semi-log plots (log_10_[x+1]) for sediment density (a) and sediment porosity (b) in relation to % C_org_ for the *Z*. *marina* sites. The sediment density (g DW mL^-1^) was negatively influencing the amount of organic carbon (R^2^ = 0.84, *P* < 0.001), while there was a positive relation between sediment porosity (%) and % C_org_ (R^2^ = 0.80, *P* < 0.001).

The amount of sediment particles < 0.074 mm (%) was significantly higher in *Z*. *marina* meadows compared to unvegetated areas (*P* < 0.001, Table 2). This was true for all four areas when pairwise comparing seagrass meadows to respective unvegetated areas (*P* < 0.05). Sediment grain size particles < 0.074 mm (%) were significantly different among areas (*P* < 0.001), where the Gullmar Fjord and Ria Formosa showed significantly higher values compared to the other areas (*P* < 0.001, Table 3), while there were no significant differences between Askö and Sozopol. There was no difference among areas in grain size particles < 0.074 mm (%) in terms of sediment depth (Table 2). Mean grain size (ɸ) and sediment particles < 0.074 mm (%) both showed strong positive linear relationship with % C_org_ in *Z*. *marina* meadows (mean grain size (ɸ), R^2^ = 0.74, *P* < 0.001; sediment particles < 0.074 mm (%), R^2^ = 0.91, *P* < 0.001, Fig 5A and 5B). For unvegetated areas, mean grain size (ɸ) did not show any relationship with % C_org_ (linear regression, R^2^ = 0.009, *P* < 0.40, Fig 5C) but was positively related to sediment particles < 0.074 mm (%) (linear regression, R^2^ = 0.42, *P* < 0.001, Fig 5D). The sediment density (g DW mL^-1^) had a negative effect on % C_org_ in the seagrass sites (linear regression, R^2^ = 0.84, *P* < 0.001) and sediment porosity (%) was positively related to % C_org_ (linear regression, R^2^ = 0.80, *P* < 0.001, Fig 6A and 6B). There was no significant relationship between % C_org_ and sediment density (g DW mL^-1^) in unvegetated areas, while sediment porosity (%) was significantly influencing % C_org_ but showed a low R_2_-value (linear regression, R^2^ = 0.08, *P* < 0.001, S2 Fig).

**Fig 7:**
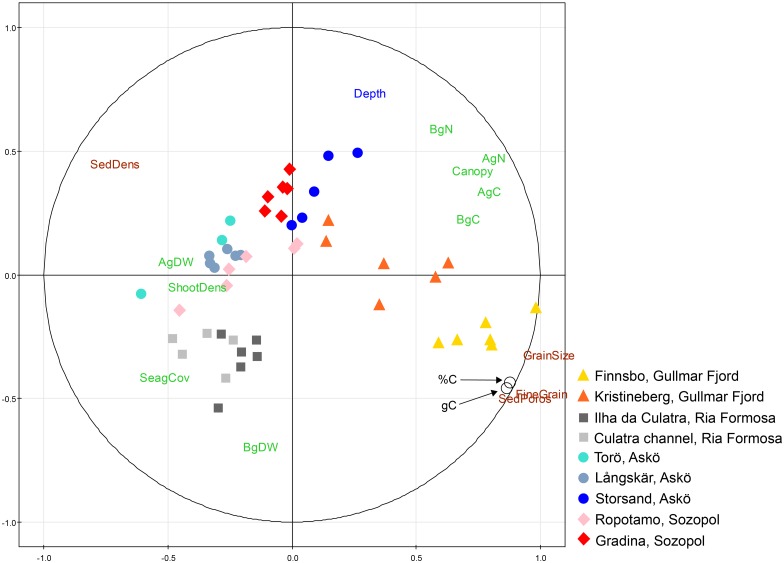
Principal component analysis (PCA) showing the nine seagrass sites, the two response variables (sedimentary % C_org_ and g C_org_ cm^-2^) and predictor variables (14 in total). The percent organic carbon (% C_org_) is presented as a mean of the content of the top 25 cm sediment, while carbon per unit area (g C_org_ cm^-2^) is the total (accumulated) amount of carbon in the top 25 cm of sediment. The colors of the letters represent different groups of predictor variables; brown letters = sediment characteristics, green letters = seagrass-associated variables, blue letters = water depth. Black circles are the response variables, i.e. organic carbon (%C = % C_org_ and gC = g C_org_ cm^-2^). For explanations to the abbreviations of predictor variables see Fig 4.

The sedimentary organic carbon content relationship to the different predictor variables was not uniform among sites. In a PCA model, the sites at the Gullmar Fjord and Ria Formosa were grouped separately from the other sites, while the Baltic- and Black Seas’ sites overlapped each other (Fig 7). The PCA model explained a large part of the variation with eigenvalues of 0.44 for PC1 and 0.25 for PC2. For the fine grain size seagrass sites of the Gullmar Fjord, the sediment characteristics (i.e. sediment particles < 0.074 mm (%), sediment porosity (%) and mean grain size (ɸ)) were important for the carbon content while the sedimentary carbon in Ria Formosa was more related to seagrass cover (%) and dry weight belowground biomass (g m^-2^). The sedimentary organic carbon content in seagrass sites in the Baltic- and Black Seas were also more related to the seagrass-associated variables, such as dry weight aboveground biomass (g m^-2^) and shoot density (shoots m^-2^), but also water depth (m) for one of the sites (Storsand, S).

## Discussion

In this assessment of four *Z*. *marina* areas in Europe, we found a large variation in organic carbon storage where the carbon-rich sediment of the Gullmar Fjord on the Swedish Skagerrak coast was 15 times higher compared to levels in the Baltic- and Black Seas. Along with recent studies [2,8], this study shows that the environmental conditions play an essential role in determining the carbon sink capacity. We found that sediment properties highly influence carbon storage in *Z*. *marina* meadows. The results showed that high sedimentary organic carbon coincides to high content of fine grain size, high sediment porosity and low sediment density. Seagrass meadows situated in areas characterized by these sediment properties are therefore suggested to have a high potential as natural carbon sinks.

Overall *Z*. *marina* meadows showed higher carbon content than nearby unvegetated areas (with the exception of the seagrass meadows with the lowest carbon storage), which is in line with previous studies (e.g. [1,4]) showing that the seagrass ecosystem is a significant carbon sink. The mean carbon content of the Gullmar Fjord was higher than the estimated global average level [4,5], illustrating the high carbon capacity of the area. The comparison with *Z*. *marina* meadows elsewhere also showed that the Swedish Skagerrak coast (e.g. the Gullmar Fjord) has an overall high carbon storage capacity (Table 5) and could potentially be a hotspot for carbon sequestration. In our study, the lowest carbon content was found in the Baltic- and Black Sea, and the Baltic Sea also had the lowest values in the literature survey (Table 5). This could be related to less suitable physical conditions of the Brackish environment with lower salinity, which may negatively affect plant growth and meadow productivity [40], in combination with growing in more exposed areas with coarser (sandy) sediment, as seen in the *Z*. *marina* meadows at Askö, where the most sheltered bays with finer grain sizes are dominated by brackish water plants, such as *Potamogeton pectinatus* and *Zannichellia palustris* [55]. Meadows situated in more exposed areas could result in a high export of the produced organic matter, as suggested by [18] instead of the carbon being accumulated in the sediment, leading to a low carbon storage potential of the area. This could also be true for the meadows in Ria Formosa, the only area in this study with a pronounced tide, where the higher hydrodynamic forces could also lead to increased sediment erosion. Although the meadows at Sozopol and Askö were dominated by *Z*. *marina* also small-sized seagrass species (i.e. *Zostera noltii* and *Ruppia maritima*) were found in minor proportion of the meadows; smaller species with lower canopy and belowground biomass could also be part of the explanation to lower sedimentary carbon concentrations as trapping of suspended particles [13] and the belowground biomass production contribute to the accumulation of carbon [15]. The trapping of fine-grained particles and prevention of sediment particle resuspension (by reducing the water velocity) in the canopy are also likely the reasons why the *Z*. *marina* meadows had substantially higher amount of smaller grain size particles compared to the unvegetated areas. Due to the fact that core shortening was not corrected for in our sediment samples there might be a margin of error up to 8% in our data. The influence of compression is most likely highest in the Skagerrak area, where the sediment is soft and has a high porosity [49], but given the large variation in carbon storage a reduction of 8% in sedimentary carbon content will not undermine our general conclusion.

**Table 5:** Summary of literature data on organic carbon (% C_org_) and organic matter (% OM) content in *Z*. *marina* sediment. In studies were only % OM was presented a conversion factor of 0.43 was used to convert % OM to % C_org_ as calculated by Fourqurean et al. [5] for seagrass sediment with > 0.2% OM. All studies have determined % OM and % C_org_ by LOI (Loss on ignition) or using an organic elemental analyzer except ^a^ where dichromate titration was used [90].

Area	Countries	Longitude (N)	Sediment core depth (cm)	C_org_ (%) ± SD	OM (%) ± SD	Water depth (m) ± SD	Sites^1^	References
Norwegian Sea	Norway	63°21'	0–10	0.49^a^		8.0	1	[77]
Skagerrak	Sweden	57°48'-59°00'	0–5	5.67 ± 3.92^+^	25.2*	2.0 ± 0.4	50	[78*,^79^]
	**Sweden**	**58°14'-58°17'**	**0–5**	**2.79 ± 2.08**		**2.9 ± 0.9**	**2**	**This study**
	Norway		0–10	0.68 ± 0.38^a^		4.0	3	[77]
Kattegat/Öresund	Denmark	54°58'-56°49'	0–5	1.68 ± 2.05^+^	4.13 ± 5.14*	2.7 **±** 0.10	11	[18*,^80^]
			0–15	0.47 ± 0.55^+^	1.26 ± 1.41	2.3 ± 2.0	6	[81–84]
Baltic Sea	Sweden, Finland	55°23'-60°21'	0–5	0.46 ± 0.30^+^	1.07 ± 0.69	2.3 ± 0.7	13	[18,43,78]
	**Sweden**	**58°49'**	**0–5**	**0.18 ± 0.04**		**3.8 ± 1.1**	**3**	**This study**
	Sweden, Finland	55°23'-60°21'	8–10	0.36 ± 0.30^+^	0.86 ± 0.70	3.3 ± 0.9	14	[33]
North Sea	Netherlands	51°34'-53°25'	0–5	0.73		Intertidal	1	[80]
			0–20	0.90 ± 0.60^+^	2.10 ± 1.40	-	5	[70]
**Black sea**	**Bulgaria**	**42°19**'**-42°22**'	**0–5**	**0.17 ± 0.07**		**4.2 ± 1.6**	**2**	**This study**
Black Sea	Bulgaria	42°24'	0–7	1.02	2.36	3.5 ± 0.9	1	[35]
North Atlantic Ocean	France, USA	34°43'-44°42'	0–5	1.78 ± 2.24^+^	4.59 ± 5.60*	2.1 ± 0.9	9	[83,85–86*, 87–88]
	USA		11–21	2.08		Intertidal	1	[87]
	**Portugal**	**36°59'-37°00'**	**0–5**	**0.61 ± 0.26**		**1.5 ± 0.6**	**2**	**This study**
Yellow Sea	China	37°20'	0–5	1.0		0.2	1	[64]
Mediterranean	Spain	36°44'	0–5	0.90^+^	2.30	13	1	[89]

^a^Dichromate titration method.

^1^Number of meadows for each area and sediment core depth.

*Studies presenting % OM if both % C_org_ and % OM are included on the same row.

^+^Converted values (partly or all) from % OM to % C_org_ (conversion factor: 0.43)

A high carbon content in *Zostera marina* sediment seems to be related to the sediment characteristics of the area. A high proportion of finer grain size particles leads to preservation and accumulation of organic matter [17,56,57] due to a higher surface area on fine-grained particles [58]. Finer grain sizes in combination with high organic matter and nutrient content, as seen in the Gullmar Fjord sites, could cause a depletion of oxygen in the sediment because of increased oxygen consumption by detritivore organisms and decreased permeability [59,60], which slows down the degradation process of organic matter [61]. In oxic conditions the bacterial communities can have 10–100 times higher degradation rate than in anoxic sediments [62]. Microbial degradation efficiency is also dependent on temperature [23] and bioturbation (leading to bioirrigation) [63]. In addition, a lower C:N ratio in the sediment reflects a higher bioavailability of organic matter promoting microbial degradation [64], and as the Gullmar Fjord showed the highest C:N ratio this could indicate an older, more refractory organic matter pool [27]. Moreover, due to a lower annual average temperature in higher latitudes, the Swedish Skagerrak and Baltic Sea coasts may show a lower degradation rate of organic matter.

Sediment grain size has recently been described as a strong predictor for carbon storage in saltmarshes [65]. For seagrass meadows, the finer grain-sized particles have shown to influence sedimentary carbon content in some seagrass areas [18], while in others it seems less important [8]. The relations between carbon storage and various sediment characteristics are more pronounced in meadows with low seagrass biomass and high proportion of finer particle sizes, while in meadows with larger seagrass species, e.g. *Posidonia* spp. and *Amphibolis* spp., having high amount of autochthonously derived sedimentary carbon, the mud and silt content has been shown to have little influence [19]. Compared to *Posidonia* spp. and *Amphibolis* spp. the smaller-sized *Z*. *marina* plants will potentially contribute less to the sediment organic matter pool, which might be the reason to why the proportion of fine sediment particles was strongly coupled to a high carbon content in the present study. Other factors have previously shown to be of importance, such as water depth, meadow productivity, sedimentation rate, trapping of fine-grained sediment and organic matter [9], and while these factors were not seen or accounted for in this study they may also be relevant when determining areas of high carbon storage potential. The grain size is directly linked to the sediment porosity and density where the organic carbon has a negative effect on sediment density [66,67]. This was also seen in our study as higher sedimentary carbon values were found in areas with lower sediment density (and hence higher porosity). For these reasons, we suggest that sediment characteristics of the area where *Z*. *marina* meadows are situated is relevant for determining the carbon storage potential.

High canopy height, high shoot density and shallow depths are generally considered to increase sedimentation rates and thus promote accumulation of finer grain size particles [13,68,69]. This implies that aboveground seagrass structure and water depth should influence the sediment carbon storage, however, in our study these variables were of minor influence. The influence of seagrass meadow structure on sediment composition is complex and hard to predict, and may be highly influenced by environmental conditions [70]. The carbon storage in *Z*. *marina* meadows in our study was clearly related to sediments with high proportion of fine grain size particles, high porosity and low density. In areas with less fine-sized sediment particles other variables, such as above- and below-ground seagrass biomass, seagrass cover and shoot density, have a more pronounced influence on carbon storage levels. For example, the influence of belowground biomass and seagrass cover on sedimentary carbon content in Ria Formosa could be due to the stabilizing properties of dense meadows [11], the binding of sediment by the root-rhizome system [71] and the high lignin content of the belowground biomass [14], which results in more decay-resistant carbon and a slower decomposition [72,73]. Seagrass biomass and cover are generally highly dynamic and act on a shorter time-scale than the sedimentary carbon storage processes, therefore estimates of present seagrass meadow properties may not be fully representative over decades or centuries, which is the likely time-scale for carbon storage in the sediment. The age of the sediment and the rate of accumulation of organic matter are factors that vary between sites where a higher sedimentation rate increases the amount of organic carbon and could be a potential explanation to variation in carbon storage among seagrass meadows [9].

The continuous loss of seagrass areas [6] leads to a decline in natural carbon sinks [74,75], and to ensure efficient management, factors for high carbon storage capacity should be evaluated. Several environmental and seagrass-related factors have shown to be of importance, i.e. water depth [10], meadow size [76], hydrodynamics and seagrass canopy complexity [8]. In our study, the main factors related to high carbon storage were the sediment density and porosity, and amount of fine grain size particles in the sediment, whereas the seagrass-associated variables had a minor influence. Therefore, we highlight that the sediment characteristics is an important factor for a high carbon storage potential in *Z*. *marina* meadows, and should be taking into consideration (together with other relevant factors) when evaluating high priority areas for protection of efficient carbon storage *Z*. *marina* areas.

## Supporting Information

S1 Fig VIP-values (variance of importance) for independent variables used in the PLS model testing relationships to carbon content. The model assesses the relative influence of different predictors on % C_org_ in sediment (using a mean for the top 25 cm sediment). The variables are listed in the level of importance and those with VIP-values >1 (dashed line) has a significant influence on the model. Brown bars = sediment characterisitcs, green bars = seagrass-associated variables and blue bars = water depth. FineGrain (sediment particles < 0.074 mm, %), SedPoros (sediment porosity, %), SedDens (sediment density, g DW mL^-1^), GrainSize (mean grain size, ɸ), Bg and Ag DW (belowground [roots and rhizomes] and aboveground [shoots] biomass dry weight, g m^-2^), Depth (water depth, m), ShootDens (shoot density m^-2^), Ag and Bg biomass C and N (biomass carbon and nitrogen content, %), Canopy (shoot height, cm), SeagrCov (seagrass cover, %) were used as predictor variables.
(DOCX)

S2 Fig Semi-log plots (log_10_[x+1]) for sediment density (g DW mL^-1^) (a), and sediment porosity (%) (b) in relation to organic carbon content (% C_org_) for unvegetated areas. There was no significant relationship between sediment density and organic carbon. The sediment porosity was, however, positively linked to sedimentary organic carbon but had a low R^2^-value (linear regression, R^2^ = 0.08, *P* < 0.001).
(DOCX)
