# Supplementary material for: Sediment Properties as Important Predictors of Carbon Storage in Zostera marina Meadows: A Comparison of Four European Areas
Source: PLoS One. 2016 Dec 9;11(12):e0167493. doi: 10.1371/journal.pone.0167493 (PMC5147920; doi:10.1371/journal.pone.0167493)
Supplement: S1 Fig — The model assesses the relative influence of different predictors on % Corg in sediment (using a mean for the top 25 cm sediment). The variables are listed in the level of importance and those with VIP-values >1 (dashed line) has a significant influence on the model. Brown bars = sediment characterisitcs, green bars = seagrass-associated variables and blue bars = water depth. FineGrain (sediment particles < 0.074 mm, %), SedPoros (sediment porosity, %), SedDens (sediment density, g DW mL-1), GrainSize (mean grain size, ɸ), Bg and Ag DW (belowground [roots and rhizomes] and aboveground [shoots] biomass dry weight, g m-2), Depth (water depth, m), ShootDens (shoot density m-2), Ag and Bg biomass C and N (biomass carbon and nitrogen content, %), Canopy (shoot height, cm), SeagrCov (seagrass cover, %) were used as predictor variables. (DOCX) [file pone.0167493.s001.docx]

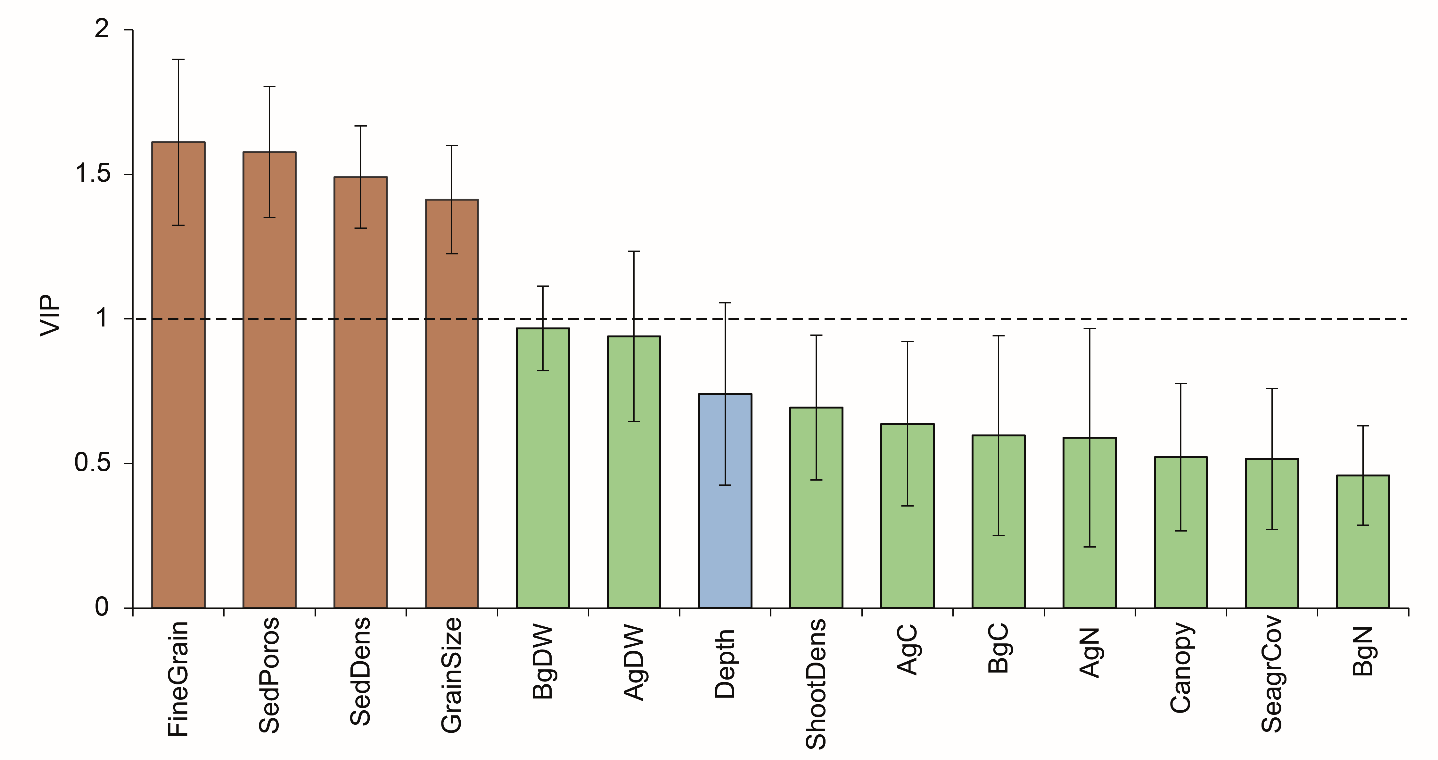


**S1 Fig. VIP-values (variance of importance) for independent variables used in the PLS model testing relationships to carbon content.** The model assesses the relative influence of different predictors on % C_org_ in sediment (using a mean for the top 25 cm sediment). The variables are listed in the level of importance and those with VIP-values >1 (dashed line) has a significant influence on the model. Brown bars = sediment characterisitcs, green bars = seagrass-associated variables and blue bars = water depth. FineGrain (sediment particles < 0.074 mm, %), SedPoros (sediment porosity, %), SedDens (sediment density, g DW mL^-1^), GrainSize (mean grain size, ɸ), Bg and Ag DW (belowground [roots and rhizomes] and aboveground [shoots] biomass dry weight, g m^-2^), Depth (water depth, m), ShootDens (shoot density m^-2^), Ag and Bg biomass C and N (biomass carbon and nitrogen content, %), Canopy (shoot height, cm), SeagrCov (seagrass cover, %) were used as predictor variables.
