# Supplementary material for: Sediment Properties as Important Predictors of Carbon Storage in Zostera marina Meadows: A Comparison of Four European Areas
Source: PLoS One. 2016 Dec 9;11(12):e0167493. doi: 10.1371/journal.pone.0167493 (PMC5147920; doi:10.1371/journal.pone.0167493)
Supplement: S2 Fig — There was no significant relationship between sediment density and organic carbon. The sediment porosity was, however, positively linked to sedimentary organic carbon but had a low R2-value (linear regression, R2 = 0.08, P < 0.001). (DOCX) [file pone.0167493.s002.docx]

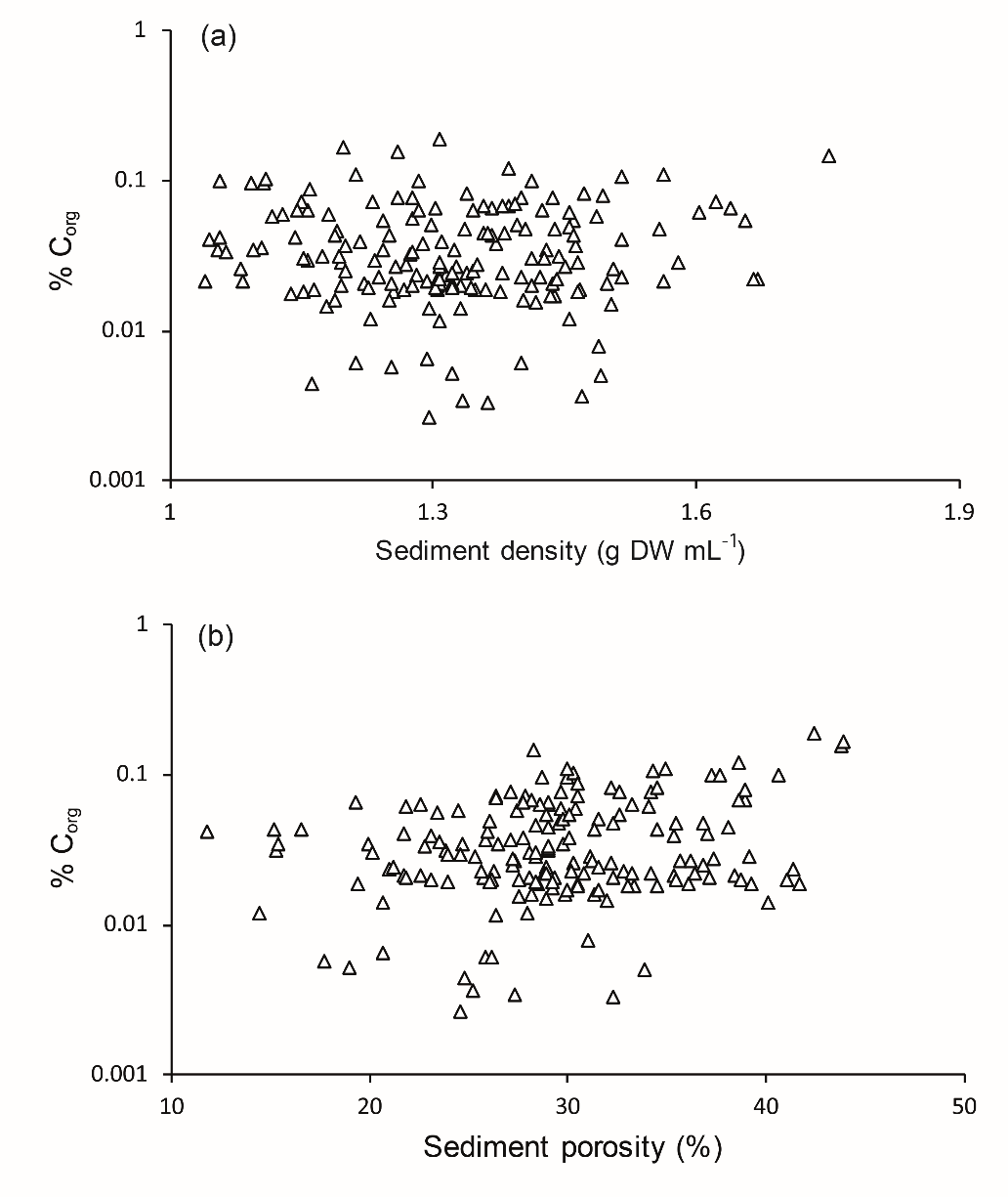


**S2 Fig.** **Semi-log plots (log_10_[x+1]) for sediment density (g DW mL^-1^) (a), and sediment porosity (%) (b) in relation to organic carbon content (% C_org_) for unvegetated areas.** There was no significant relationship between sediment density and organic carbon. The sediment porosity was, however, positively linked to sedimentary organic carbon but had a low R^2^-value (linear regression, R^2^ = 0.08, *P* < 0.001).
